# Identification of diagnostic KASP‐SNP markers for routine breeding activities in yam (*Dioscorea* spp.)

**DOI:** 10.1002/tpg2.20419

**Published:** 2023-12-13

**Authors:** Paterne A. Agre, Lindsay V. Clark, Ana Luisa Garcia‐Oliveira, Rajaguru Bohar, Patrick Adebola, Robert Asiedu, Ryohei Terauchi, Asrat Asfaw

**Affiliations:** ^1^ International Institute of Tropical Agriculture (IITA) Ibadan Nigeria; ^2^ HPCBio, Roy J. Carver Biotechnology Center University of Illinois at Urbana‐Champaign Urbana Illinois USA; ^3^ Excellence in Breeding (EiB), CIMMYT‐ICRAF, UN Av Nairobi Kenya; ^4^ Department of Molecular Biology, Biotechnology and Bioinformatics, College of Basic Sciences and Humanities CCS Haryana Agricultural University Hisar Haryana India; ^5^ Excellence in Breeding (EiB), CIMMYT‐ICRISAT Hyderabad Telangana India; ^6^ Laboratory of Crop Evolution, Graduate School of Agriculture Kyoto University Kyoto Japan; ^7^ Iwate Biotechnology Research Center Kitakami Japan

## Abstract

Maintaining genetic purity and true‐to‐type clone identification are important action steps in breeding programs. This study aimed to develop a universal set of kompetitive allele‐specific polymerase chain reaction (KASP)‐based single nucleotide polymorphism (SNP) markers for routine breeding activities. Ultra‐low‐density SNP markers were created using an initial set of 173,675 SNPs that were obtained from whole‐genome resequencing of 333 diverse white Guinea yam (*Dioscorea rotundata* Poir) genotypes. From whole‐genome resequencing data, 99 putative SNP markers were found and successfully converted to high‐throughput KASP genotyping assays. The markers set was validated on 374 genotypes representing six yam species. Out of the 99 markers, 50 were highly polymorphic across the species and could distinguish different yam species and pedigree origins. The selected SNP markers classified the validation population based on the different yam species and identified potential duplicates within yam species. Through penalized analysis, the male parent of progenies involved in polycrosses was successfully predicted and validated. Our research was a trailblazer in validating KASP‐based SNP assays for species identification, parental fingerprinting, and quality control (QC) and quality assurance (QA) in yam breeding programs.

## Core Ideas

- This study is a pioneer that validated kompetitive allele‐specific (KASP)‐based single nucleotide polymorphism (SNP) assay for quality control (QC) and assurance in yam breeding.
- We developed 50 KASP‐SNPs that are able to distinguish different yam species.
- Our quality assurance (QA) and QC markers effectively track genetic purity of breeding lines and cultivars and assess fidelity of crosses.

**Abbreviations**
APP: ancestry posterior probability
Bp: basepair
DAPC: discriminant analysis of principal components
DNA: deoxyribonucleic acid
He: expected heterozygosity
Ho: observed heterozygosity
KASP: kompetitive allele‐specific
MAF: minor allele frequency
PCA: principal component analysis
PIC: polymorphism information content
Pop: subpopulation
QA: quality assurance
QC: quality control
SNP: single nucleotide polymorphism
VCF: variant call format

## 1 INTRODUCTION

Yam (*Dioscorea* spp.) is a multi‐species monocotyledonous tuber crop predominantly cultivated in the tropical regions of Africa, Southeast Asia, and Latin America (Azeteh et al., 2019; Darkwa, Olasanmi, et al., 2020; Oben et al., 2016). *Dioscorea* encompasses about 600 species (Burkill, 1960), of which only 11 are widely cultivated species (Darkwa, Olasanmi, et al., 2020). The major cultivated species have basic chromosome number *X* = 20 (Scarcelli et al., 2019) but have variable ploidy (2*n* = 40, 60, 80) between and within the species (Sugihara et al., 2020). Among the cultivated species, water/purple yam (*Dioscorea alata* L.) is the most extensively distributed species globally from the forest to grassland in the tropical and temperate regions. In contrast, white Guinea yam (*Dioscorea rotundata* Poir) is the most widely planted and produced yam globally (Asfaw et al., 2021), with its predominant cultivation coming from West Africa, a region where Nigeria, Ghana, and Cote d'Ivoire together produce nearly 86% of the world's yam supply (FAOSTAT, 2020). In West Africa, yam is a valuable food security crop, with about 400 million people depending on it as a staple food. In addition to serving as an important source of dietary carbohydrates, it also serves as a significant source of income for producers and a sociocultural asset for the society that depends on it (Aighewi et al., 2020; Nweke, 2016).

The informal nature of yam trade, sharing of germplasm among countries, and the exchange of planting materials among farmers have resulted in the same cultivar carrying different names or different cultivars carrying the same name in various yam farming systems (Agre et al., 2021; Otoo et al., 2009). Such phenomenon has also been reported in cassava (*Manihot esculenta* L.) studies (Agre et al., 2018; Rabbi et al., 2015). Furthermore, human errors such as mislabeling and material mix‐up during planting, harvesting, transportation, or storage are the leading causes of misidentification of accessions in genebank collections and breeding programs (Girma et al., 2012). Therefore, control and understanding of germplasm identity and purity, as well as hybrid authentication, are essential action steps for breeding efficiency management.

Single nucleotide polymorphisms (SNPs) are vital molecular markers for genetic analysis and breeding. The next‐generation sequencing technique, which generates millions of SNP markers, has provided a faster and more affordable opportunity to employ genotyping as a low cost and robust component of routine breeding activities (Chen et al., 2016; Semagn et al., 2014). However, in yam, the majority of molecular studies have been focused on de novo sequencing (Saski et al., 2015), the development of reference genome (Sugihara et al., 2020; Tamiru et al., 2017), genetic diversity analysis (Agre et al., 2019, 2021; Akakpo et al., 2017; Darkwa, Agre, et al., 2020; Girma et al., 2014, 2015; Loko et al., 2017; Siadjeu et al., 2018), evolution of yam domestication (Sugihara et al., 2020), quantitative trait locus identification (Agre et al., 2022; Bhattacharjee et al., 2018; Cormier, Lawac, et al., 2019), and genomic prediction (Norman et al., 2020).

**Core Ideas**

- This study is a pioneer that validated kompetitive allele‐specific (KASP)‐based single nucleotide polymorphism (SNP) assay for quality control (QC) and assurance in yam breeding.
- We developed 50 KASP‐SNPs that are able to distinguish different yam species.
- Our quality assurance (QA) and QC markers effectively track genetic purity of breeding lines and cultivars and assess fidelity of crosses.

Routine breeding activities (assessment of genetic diversity or identification of mislabeled genotypes, pedigree verification, variety tracking, so on), also known as quality control (QC) in crop breeding leveraging molecular breeding tools, have been employed as a routine practice in many breeding and seed programs to harness genetic gain and delivery of quality products to the end users. These practices have been developed and deployed in several crops, including maize (*Zea mays* L.) (Chen et al., 2016; Gowda et al., 2017; Semagn et al., 2012), rice (Oryza sativa) (Ndjiondjop et al., 2018), sweetpotato (*Ipomoea batatas* (L.) Lam) (Gemenet et al., 2020), cowpea (*Vigna unguiculata* (L.) Walp.; Ongom et al., 2021), and soybean (*Glycine max*; Chander et al., 2021). Except for a recent study by Cormier, Mournet, et al. (2019) that presented kompetitive allele‐specific (KASP)‐based SNP markers for ploidy evaluation in water/purple yam, efforts to establish QC markers for yam are scanty. In order to examine the genetic purity of breeding lines at testing stages and cultivars in seed systems, as well as to authenticate the hybridity of offspring from crosses, the goal of this project was to develop a universally applicable, highly informative quality control/assurance (QC/QA) markers set.

## 2 MATERIALS AND METHODS

### 2.1 Plant materials used for generating raw SNP markers

In this study, three sets of materials were used. Set 1 consisted of 333 yam clones encompassing breeding lines, landrace cultivars, and gene bank accessions using whole‐genome resequencing to identify high polymorphic SNP markers.

Set 2 materials composed of 374 yam clones representing six different species (*D. alata*, *D. bulbifera*, *D. cayenensis*, *D. praehensilis*, *D. esculenta*, and *D. rotundata)*. The materials were used for high‐throughput KASP assay design and marker validation. Dataset 3 consisted of 27 yam clones, including four putative parents (one female and three males), and their 23 progenies originated from unsupervised polycross involving these four parents at the International Institute of Tropical Agriculture yam breeding.

### 2.2 Sequencing of dataset 1

Total genomic deoxyribonucleic acid (DNA) from dataset 1 was extracted following the protocol described by Tamiru et al. (2017). DNA library construction and whole‐genome resequencing were conducted using the protocol described by Sugahira et al. (2020). Generated reads were aligned with yam reference genome v2 (Sugahira et al., 2020) using Genome Analysis Toolkit v3.8‐0 to generate a variant call format file (VCF). The VCF was later filtered for minor allele frequency (MAF), missing data (both at genotypes and SNP markers level), polymorphism information content, heterozygosity, and read depth.

### 2.3 Selection of highly polymorphic SNP markers

SNP markers were then further filtered using a custom R script to retain only those that had a ratio of observed to expected heterozygosity (He) of less than 1.5, a guanine‐cytosine content of 40%–60% in the 101 bp window extending 50 basepairs (Bps) on either side of the SNP, and two or fewer flanking SNPs in that same window. Flanking SNPs were ignored if their quality score was 900 or lower but were not otherwise subjected to filtering in PLINK or R. Two approaches were then used for selecting SNPs. In the first approach, the Purity tool in the Excellence in Breeding Galaxy instance (http://cropgalaxy.excellenceinbreeding.org/root?tool_id = purity_beta) was used to identify a set of 50 markers that best distinguished all individuals in dataset 1. In the second approach, the dataset was subdivided into nine populations using discriminant analysis of principal components (DAPC) (Jombart et al., 2010) and run against a simulated annealing algorithm developed in R to find a set of 50 markers that maximized diversity within and between these nine populations. Using these two methods, 99 markers were selected in total. The process of the SNP selection, including all R codes, is documented and available online via the following link: https://github.com/HPCBio/eib‐marker‐design/blob/main/Pedigree_verification.md, archived on Zenodo at https://doi.org/10.5281/zenodo.6093835.

### 2.4 Design, verification, and validation of KASP markers from selected SNP sequences

Flanking sequences surrounding each selected SNP (50 bp at both upstream and downstream around the variant position) were generated using a custom R script, with any flanking SNPs coded using International Union of Pure and Applied Chemistry ambiguity codes. The flanking sequences for the 99 selected SNPs were then submitted for primer synthesis, verified, and validated on a panel of 374 yam accessions composed of dataset 2 and 3 (https://yambase.org/pages/markers).

### 2.5 Data analysis

Various population genetic analyses were conducted to explore the genetic properties of the markers. DAPC were generated first using 136,429 SNPs and then with the selected 99 discriminative SNP markers through Adegenet library package in R (Jombart et al., 2010), and the membership probabilities were compared. For dataset 2, Hapmap and VCF files were generated for the raw data. Summary statistics, such as missing values, MAF, heterozygous frequency (observed and expected), polymorphism information content (PIC), and discriminant markers, were accessed using PLINK (Purcell et al., 2007) and VCFTOOLS. SNP markers were then classified based on the level of the polymorphism information content, their ability to discriminate yam species, and their contribution to identifying true‐to‐type yam clones. The most polymorphic SNP markers were selected and used to assess genetic diversity through principal component analysis (PCA) and hierarchical cluster analysis. To understand the frequency of the different recombinations in dataset 2, admixture analysis was conducted, whereby clones with ancestry posterior probability (APP) >0.5 were assigned to a group while clones with (APP) <0.5 were considered as admixed. Intentional duplicate clones were used to evaluate the accuracy of some selected SNP markers in detecting potential duplicates. Twenty‐three progenies with four putative parents (one female and three males) used in previous pedigree estimation by Norman et al. (2020) were incorporated in the present study for pedigree reconstruction. The Penalized and doMC libraries were used to estimate the contribution of the male and female parents to the offspring (McIlhagga, 2016). The value of each parent was estimated using the multinomial model of the penalized function formula as previously described (McIlhagga, 2016; Norman et al., 2020) and the final pedigree was visualized using the Helium visualization tool (Shaw et al., 2014).

## 3 RESULTS

### 3.1 Summary statistics of the selected SNP markers

Using dataset 1, 99 SNPs were identified as informative and discriminative. The selected SNP markers were successfully converted into KASP markers on 374 yam accessions (dataset 2). Considering the total SNP markers and across all the species, an average value of 0.29 was obtained for the MAF and the lowest average value was recorded in *D. esculenta* species (Table 1). For the polymorphism information content, the average value recorded was 0.29, with the lowest value recorded in *D. esculenta* (Table 1). For the observed heterozygosity (Ho), it varied from 0.17 in *D. esculenta* to 0.37 in *D. rotundata*, with the remaining subspecies presenting in‐between values.

**TABLE 1:** Genetic statistic summary of the 99 single nucleotide polymorphism (SNP) markers based on dataset 2.

Genetic parameters	Across species	*D. rotundata* (*n* = 140)	*D. praehensilis* (*n* = 43)	*D. cayenensis* (*n* = 25)	*D. alata* (*n* = 120)	*D. esculenta* (*n* = 20)	*D. bulbifera* (*n* = 26)
APIC	0.29	0.29	0.12	0.12	0.14	0.08	0.11
MAF	0.29	0.31	0.16	0.16	0.16	0.10	0.13
He	0.37	0.38	0.16	0.16	0.18	0.10	0.15
Ho	0.32	0.37	0.32	0.31	0.29	0.17	0.23
NA	0.00	0.00	0.01	0.04	0.26	0.73	0.37

Abbreviations: APIC, average polymorphism information content; He, expected heterozygosity; Ho, observed heterozygosity; MAF, minor allele frequency; NA, missing proportion; n, number of genotypes.

Based on the different genetic parameters (PIC, MAF, Ho, He, and the proportion of the missing information), 50 SNP markers of the 99 tested were identified with good genetic parameters information (Table 2). Additional information related to the SNP makers, such as the flanking sequencing and the alleles variant can be freely downloaded from the following link: https://figshare.com/articles/dataset/List_of_50_SNP_markers/24072339.

**TABLE 2:** List of the 50 highly informative single nucleotide polymorphism (SNP) markers selected for routine breeding activities.

SN	SNP ID	Chro	Pos	Ref All	Alt All	Additional information and flanking
1	snpDR00108	1	27275023	*T*	*C*	^https://figshare.com/account/items/24072339/edit^
2	snpDR00135	2	16909288	*T*	*C*	^https://figshare.com/account/items/24072339/edit^
3	snpDR00120	2	23698309	*T*	*G*	^https://figshare.com/account/items/24072339/edit^
4	snpDR00136	3	18568582	*T*	*C*	^https://figshare.com/account/items/24072339/edit^
5	snpDR00138	4	10618167	*A*	*G*	^https://figshare.com/account/items/24072339/edit^
6	snpDR00145	4	8900737	*A*	*G*	^https://figshare.com/account/items/24072339/edit^
7	snpDR00137	5	1384592	*A*	*C*	^https://figshare.com/account/items/24072339/edit^
8	snpDR00056	5	15642980	*G*	*C*	^https://figshare.com/account/items/24072339/edit^
9	snpDR00118	6	10055883	*G*	*A*	^https://figshare.com/account/items/24072339/edit^
10	snpDR00059	6	1389600	*T*	*G*	^https://figshare.com/account/items/24072339/edit^
11	snpDR00062	6	16474865	*C*	*T*	^https://figshare.com/account/items/24072339/edit^
12	snpDR00065	7	25862177	*C*	*T*	^https://figshare.com/account/items/24072339/edit^
13	snpDR00107	7	29826812	*C*	*A*	^https://figshare.com/account/items/24072339/edit^
14	snpDR00066	8	14532723	*T*	*C*	^https://figshare.com/account/items/24072339/edit^
15	snpDR00128	8	16065388	*A*	*C*	^https://figshare.com/account/items/24072339/edit^
16	snpDR00123	9	6928209	*T*	*C*	^https://figshare.com/account/items/24072339/edit^
17	snpDR00112	10	10660488	*G*	*T*	^https://figshare.com/account/items/24072339/edit^
18	snpDR00139	10	11528458	*A*	*G*	^https://figshare.com/account/items/24072339/edit^
19	snpDR00109	10	14200449	*T*	*C*	^https://figshare.com/account/items/24072339/edit^
20	snpDR00069	10	23384975	*T*	*G*	^https://figshare.com/account/items/24072339/edit^
21	snpDR00124	10	272561	*G*	*T*	^https://figshare.com/account/items/24072339/edit^
22	snpDR00115	10	663980	*G*	*A*	^https://figshare.com/account/items/24072339/edit^
23	snpDR00142	11	13649874	*C*	*T*	^https://figshare.com/account/items/24072339/edit^
24	snpDR00071	11	15371387	*T*	*C*	^https://figshare.com/account/items/24072339/edit^
25	snpDR00075	12	19554292	*C*	*T*	^https://figshare.com/account/items/24072339/edit^
26	snpDR00076	12	19646887	*A*	*C*	^https://figshare.com/account/items/24072339/edit^
27	snpDR00146	14	20907274	*G*	*A*	^https://figshare.com/account/items/24072339/edit^
28	snpDR00079	14	8004400	*A*	*G*	^https://figshare.com/account/items/24072339/edit^
29	snpDR00149	15	11969634	*A*	*G*	^https://figshare.com/account/items/24072339/edit^
30	snpDR00132	15	12971805	*C*	*T*	^https://figshare.com/account/items/24072339/edit^
31	snpDR00129	15	13236354	*A*	*C*	^https://figshare.com/account/items/24072339/edit^
32	snpDR00084	15	13652946	*C*	*T*	^https://figshare.com/account/items/24072339/edit^
33	snpDR00114	15	14027608	*A*	*G*	^https://figshare.com/account/items/24072339/edit^
34	snpDR00111	15	16810337	*T*	*C*	^https://figshare.com/account/items/24072339/edit^
35	snpDR00140	15	17159846	*C*	*T*	^https://figshare.com/account/items/24072339/edit^
36	snpDR00082	15	3532953	*A*	*C*	^https://figshare.com/account/items/24072339/edit^
37	snpDR00088	16	11941748	*T*	*C*	^https://figshare.com/account/items/24072339/edit^
38	snpDR00090	16	18591145	*A*	*G*	^https://figshare.com/account/items/24072339/edit^
39	snpDR00125	16	20344737	*A*	*G*	^https://figshare.com/account/items/24072339/edit^
40	snpDR00091	16	22290712	*G*	*C*	^https://figshare.com/account/items/24072339/edit^
41	snpDR00094	17	12293584	*C*	*T*	^https://figshare.com/account/items/24072339/edit^
42	snpDR00096	17	19171588	*T*	*G*	^https://figshare.com/account/items/24072339/edit^
43	snpDR00143	17	19787441	*T*	*C*	^https://figshare.com/account/items/24072339/edit^
44	snpDR00092	17	2594646	*G*	*C*	^https://figshare.com/account/items/24072339/edit^
45	snpDR00093	17	2672794	*G*	*C*	^https://figshare.com/account/items/24072339/edit^
46	snpDR00097	18	15163101	*T*	*G*	^https://figshare.com/account/items/24072339/edit^
47	snpDR00106	19	21300319	*G*	*A*	^https://figshare.com/account/items/24072339/edit^
48	snpDR00147	19	22363327	*A*	*G*	^https://figshare.com/account/items/24072339/edit^
49	snpDR00099	19	22645412	*A*	*G*	^https://figshare.com/account/items/24072339/edit^
50	snpDR00100	19	22953072	*C*	*T*	^https://figshare.com/account/items/24072339/edit^

Abbreviations: Alt All, alternative allele; Chr, chromosome; Pos, physical marker position; Ref All, reference allele.

### 3.2 Population structure, species and variety identification, and germplasm management

**FIGURE 1:**
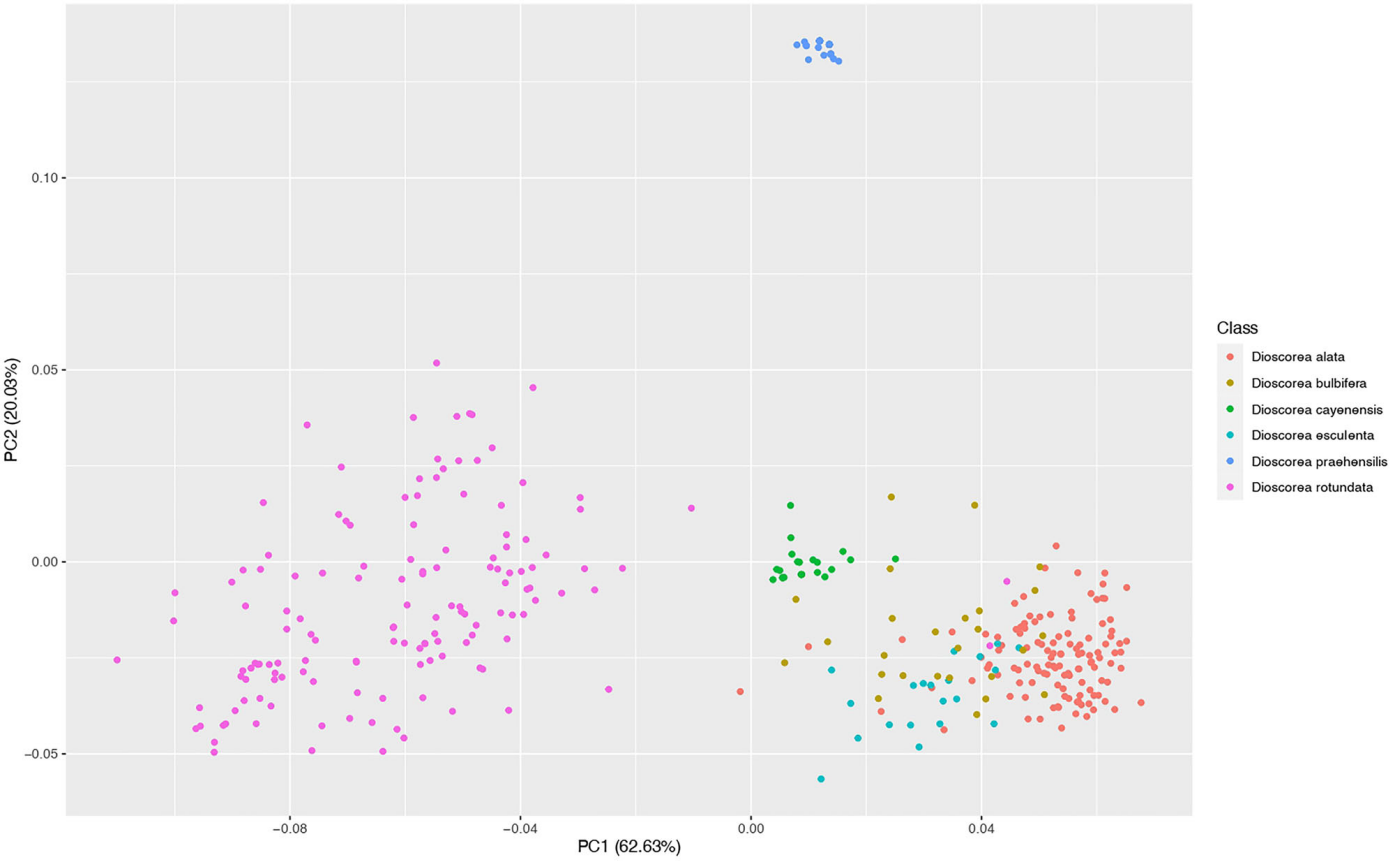
Principal component analysis of 374 yam accessions (dataset 2) using 50 single nucleotide polymorphism (SNP) markers. The different colors represent the different species used in the study.

**FIGURE 2:**
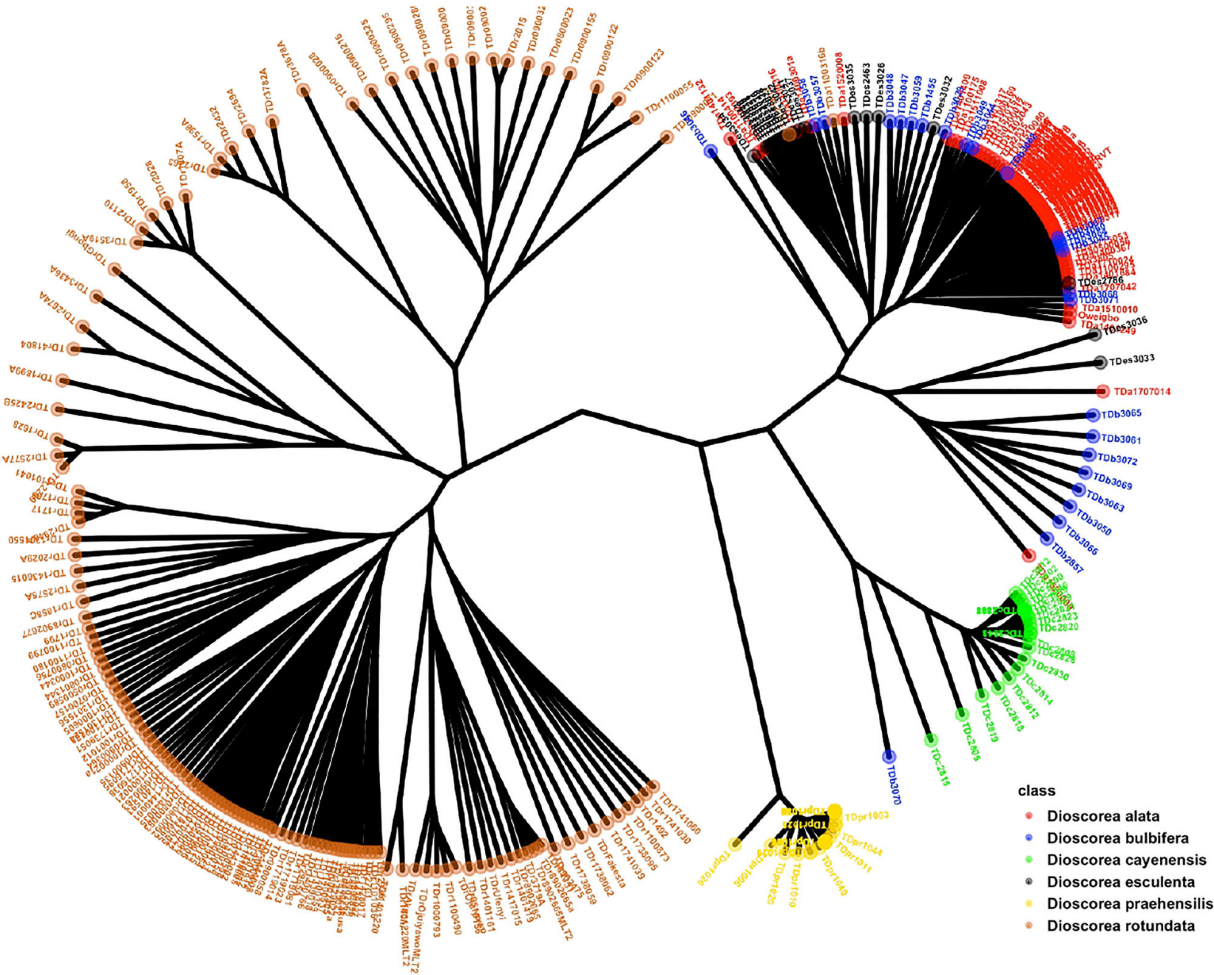
Hierarchical clustering using a set of 50 single nucleotide polymorphism (SNP) markers on 374 yam accessions. Each color represents a cluster containing accessions of different species.

The 50 highly polymorphic SNP markers selected were used to genetically characterize 374 yam accessions consisting of different yam species (*D. rotundata, D. praehensilis*, *D. cayenensis*, *D. alata, D. esculenta* and *D. bulbifera*) utilizing PCA and hierarchical clustering. The first two components of the PCA accounted for 82.6% of the total variation among the accessions (Figure 1). The PCA revealed clear separation among the different yam species involved in this study and identified *D. alata, D. esculenta*, *and D. bulbifera* to be closer than the rest of the species (Figure 1). Based on the identity‐by‐state matrix (dissimilarity matrix), the genetic distance varied from 0.01 to 0.59, with the lowest value obtained among accessions of the same species. In contrast, the highest genetic distance was observed between *D. rotundata* and *D. alata*. Hierarchical clustering revealed the presence of four major clusters (Figure 2). Cluster 1 had only *D. rotundata* (cyan color*)*, cluster 2 only *D. praehensilis* (yellow color), cluster 3 (green color) had only *D. cayenensis*, while cluster 4 (red, black, and blue) is a combination of three different species *(D. alata, D. esculenta*, and *D. bulbifera)*. Cluster 1 consisted of only *D. rotundata* (138 in total) with several intentional duplicated clones (Figure 2). The local varieties, such as Hemba and Pampar, previously identified as duplicates using high‐density SNP markers, were herein confirmed as duplicates using this QC/QA marker set. In cluster 2, made of only *D. praehensilis*, the genetic distance varied from 0.01 to 0.22, with many clones identified as closely related (Figure 2). In cluster 3, only *D. cayenensis* was grouped in that cluster and displayed a very low genetic distance among the studied accessions. Cluster 4 had the largest membership, with three different species, namely *D. alata, D. esculenta*, and *D. bulbifera*.

**FIGURE 3:**
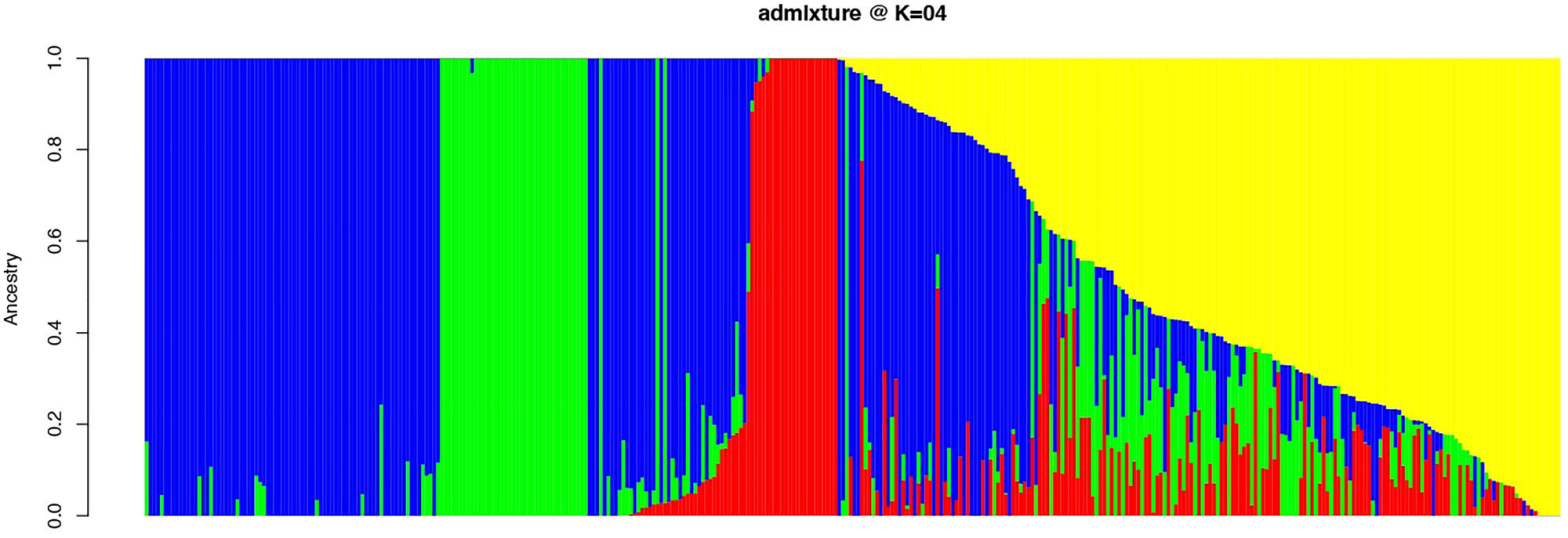
Admixture results using the 374 yam accessions (dataset 2) and the 50 single nucleotide polymorphism (SNP) markers. Each different color represents a group of clones while each bar plot is an accession.

Admixture was used to assess the population (dataset 2). The *K*‐value was used to estimate the accession cluster number based on the 50 SNP markers. To determine the optimal *K*‐value, the cluster number (*K*) was plotted against Δ*K*, which showed a sharp elbow at *K* = 4 (Figure S1). The optimal *K*‐value indicates that four subpopulations (Pop1, Pop2, Pop3, and Pop4) showed the highest probability of population clustering (Figure 3). In addition, and across the four Pops, few accessions were identified with ancestry probability greater than 50%, and such accessions were identified as no admixt. Based on the ancestor probability of assignment, the highest admixed clones’ rate was recorded in the *D. rotundata* breeding lines.

### 3.3 Pedigree reconstruction of *D. rotundata* using 50 SNP markers

**FIGURE 4:**
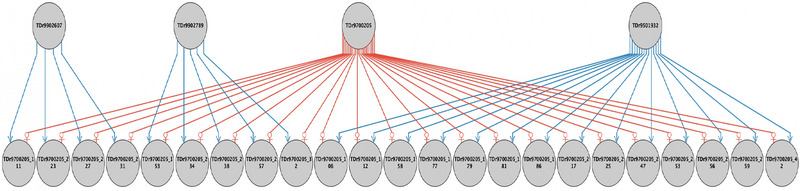
Visual display of the pedigree reconstruction with the selected highly informative 50 single nucleotide polymorphism (SNP) markers on the 23 *D.rotundata* breeding lines derived from the four parents in unsupervised polycross design.

The paternity of 23 progenies originated from a polycross between a single female (TDr9700205) and three different males (TDr9501932, TDr9902789, and TDr9902607), which was reconstructed. Through the penalized analysis and the pedigree reconstruction, it was ascertained that male TDr95011932 contributed to the highest (61%) pollination for most of the progenies (Figure 4).

## 4 DISCUSSION

We developed a subset of a few and highly informative SNP markers for routine use in breeding and seed programs for assessing the genetic purity of breeding lines and released cultivars and hybridity of progenies from crosses. The recent advances in molecular technology have emphasized on the use of SNP markers because they are cost effective, adequately abundant in the genome, locus‐specific, codominant, and have the potential for high throughput compared to other markers (Hamblin et al., 2007; Josia et al., 2021). The use of many markers is recommended in the initial steps of understanding the genetic material that a breeding program possesses, yet in a later stage, it is highly recommended to use a smaller and more effective number of markers for cost‐effectiveness (Cormier, Mournet, et al., 2019; Islam et al., 2015; Ramakrishnan et al., 2015; Vos et al., 2015). The utilization of very few informative SNP markers for routine breeding activities without compromising the original results has been established in many crops such as chickpea (*Cicer arietinum* L.) (Hiremath et al., 2012), potato (*Solanum tuberosum*) (Vos et al., 2015), cotton (*Gossypium herbaceum*; lslam et al., 2015), maize (Chen et al., 2016), rice (Ndjiondjop et al., 2018), as well as soybean (Chander et al., 2021). In a similar study, using 40 water/purple yam accessions, Cormier, Mournet, et al. (2019) identified 4593 genome‐wide SNP markers, out of which 129 representative SNPs came out as KASP markers for ploidy level assessment in water/purple yam. In our case, out of 136,429 SNPs assayed, 99 were successfully developed and validated on different yam species accessions and allowed the selection of highly informative 50 polymorphic SNPs characterized by having a high PIC, MAF, and high species discrimination rate. Hierarchical clustering and the PCA highlighted the efficiency of the subset of the selected 50 SNP markers to identify yam species and the recognition of identical clones within the different species studied. In line with our finding, Semagn et al. (2012) and Zhou et al. (2016) have reported that 50–100 singleplex assay SNPs are sufficient for molecular‐based QC genotyping. The selected 50 SNP markers herein described can be applied in diverse yam breeding programs at the lowest cost without compromising the quality of routine activities, such as pedigree verification, variety tracking, and germplasm maintenance.

## 5 CONCLUSIONS

We conducted a pilot study on the development of SNP markers for *Dioscorea* spp. and employed them for yam varietal and species identification using KASP polymerase chain reaction technology. In order to create a universal DNA fingerprint for yam, 50 highly polymorphic KASP SNP markers were chosen. The SNP panel described here offers a valuable KASP resource to aid in the genetic identification of yam germplasm and correct errors made during normal activities in breeding and seed multiplication phases. The yam community will greatly benefit from the practical applications of this study's findings.

## AUTHOR CONTRIBUTIONS

**Paterne A. Agre**: Conceptualization; formal analysis; investigation; methodology; writing—original draft; writing—review and editing. **Lindsay V. Clark**: Formal analysis; methodology; writing—original draft; writing—review and editing. **Ana Luisa Garcia‐Oliveira**: Methodology; writing—original draft; writing—review and editing. **Rajaguru Bohar**: Writing—review and editing. **Patrick Adebola**: Project administration; writing—review and editing. **Robert Asiedu**: Writing—review and editing. **Ryohei Terauchi**: Resources; writing—review and editing. **Asrat Asfaw**: Conceptualization; resources; supervision; writing—original draft; writing—review and editing.

## CONFLICT OF INTEREST STATEMENT

The authors declare no conflicts of interest.

## Supporting information


**SUPPLEMENTARY FIGURE 1**: Optimum number of cluster.

## Data Availability

The variant call format (VCF) file used for analyses of data‐sets 1–3 are all available under the genotypic project at: www.yambase.org. The list of the QC/QA markers can be downloaded through the following link: https://yambase.org/pages/markers.
